# Functional analysis of the airways after pulmonary lobectomy through computational fluid dynamics

**DOI:** 10.1038/s41598-022-06852-x

**Published:** 2022-02-28

**Authors:** Lorenzo Aliboni, Marta Tullio, Francesca Pennati, Antonella Lomauro, Rosaria Carrinola, Gianpaolo Carrafiello, Mario Nosotti, Alessandro Palleschi, Andrea Aliverti

**Affiliations:** 1 TBMLab - Laboratorio di Tecnologie Biomediche, Dipartimento di Elettronica, Informazione e Bioingegneria (DEIB), Politecnico Di Milano, Via Colombo, 40 (2° floor), 20133 Milan, Italy; 2 Department of Pathophysiology and Transplantation, University of Milan, Milan, Italy; 3 Thoracic Surgery and Lung Transplantation Unit, Fondazione IRCCS Ca’ Granda Ospedale Maggiore Policlinico of Milan, Milan, Italy; 4 Diagnostic and Interventional Radiology Department, Fondazione IRCCS Cà Granda Ospedale Maggiore Policlinico, 20122 Milan, Italy; 5 Department of Radiology and Department of Health Sciences, Fondazione IRCCS Cà Granda Ospedale Maggiore Policlinico and University of Milano, 20122 Milan, Italy

**Keywords:** Computational models, Surgical oncology

## Abstract

Pulmonary lobectomy, which consists of the partial or complete resection of a lung lobe, is the gold standard intervention for lung cancer removal. The removal of functional tissue during the surgery and the re-adaptation of the remaining thoracic structures decrease the patient's post-operative pulmonary function. Residual functionality is evaluated through pulmonary function tests, which account for the number of resected segments without considering local structural alterations and provide an average at-the-mouth estimation. Computational Fluid Dynamics (CFD) has been demonstrated to provide patient-specific, quantitative, and local information about airways airflow dynamics. A CFD investigation was performed on image-based airway trees reconstructed before and after the surgery for twelve patients who underwent lobectomy at different lobes. The geometrical alterations and the variations in fluid dynamics parameters and in lobar ventilation between the pre and post-operative conditions were evaluated. The post-operative function was estimated and compared with current clinical algorithms and with actual clinical data. The post-operative configuration revealed a high intersubject variability: regardless of the lobectomy site, an increment of global velocity, wall pressure, and wall shear stress was observed. Local flow disturbances also emerged at, and downstream of, the resection site. The analysis of lobar ventilation showed severe variations in the volume flow rate distribution, highlighting the compensatory effects in the contralateral lung with an increment of inflow. The estimation of post-operative function through CFD was comparable with the current clinical algorithm and the actual spirometric measurements. The results confirmed that CFD could provide additional information to support the current clinical approaches both in the operability assessment and in the prescription of personalized respiratory rehabilitation.

## Introduction

Anatomical pulmonary resection (i.e., segmentectomy, lobectomy, or pneumonectomy) is the cornerstone of therapy for early-stage lung cancer. Particularly, pulmonary lobectomy removes the individual affected lobe, with related loss of respiratory lung tissue. On the other hand, lobectomy induces post-operative adaptation of pleural space and chest wall. These changes occur ipsilateral (hemithoracic volume decrease, mediastinal shift, diaphragmatic rise) and contralateral (parenchymal overdistension) to the operated side^1,2^. The resulting changes of the tracheobronchial tree in the topographical anatomy and the presence of the bronchial stump itself modify the airflow and the secretion clearance^3–5^. Post-operative alterations differ according to the excised lobe^3,6^, however in the long-term after surgery, alveolar tissue regrowth, structural remodeling, and progressive functional compensation are observed^7,8^. The prediction of post-operative pulmonary function is crucial for establishing the operability of the patient and assessing the surgical risk. The current clinical practice relies on pre-operative pulmonary function tests (PFTs), i.e., spirometry and cardiopulmonary exercise tests^9^. From this information, the post-operative function is estimated using an anatomical method that accounts for the number of segments that will be resected (segment counting) or using the lung perfusion scans^10,11^. However, these methods do not account for volume differences among pulmonary segments and lobes, tissue heterogeneity, and post-operative alterations of lung and airway anatomy^12,13^.

Computational Fluid Dynamics (CFD) represents a feasible solution to overcome the drawbacks of the current clinical approaches. From image-based patient-specific bronchial tree reconstructions, CFD can provide quantitative information on regional ventilation and local airflow properties in the bronchial tree^14–17^. CFD has been applied to several pathologies of the lower respiratory tract, such as tracheal stenosis^18^, asthma, chronic obstructive pulmonary disease^19,20^, and cystic fibrosis^21^. The effects on fluid dynamics of congenital anomalies such as the left pulmonary sling have also been studied^22^. Recently, lobectomy effects in patients who underwent left upper pulmonary lobectomy have been investigated^23,24^ showing significant alterations in the geometry and fluid dynamics between the pre and post-operative models. Although several differences are reported in terms of post-operative changes among lower, middle, and upper lobectomies^3,6^, to date, the effects of lobectomy on the fluid dynamics have been analyzed only for the left upper lobectomy. Consequently, it is of primary interest to perform patient-specific CFD simulations for the remaining lobes and analyze the fluid dynamic parameters that will emerge. This will better characterize the effects of post-operative remodeling on airways fluid dynamics allowing a quantitative assessment of the pre-operative condition according to the surgery site. In addition, the information of pre-operative flow rate and local fluid dynamics characteristic of each branch can be used, without the need for post-operative data, to estimate the post-operative function. This approach will provide additional information concerning patient operability as it accounts not only for the number of segments that will be removed (based on a surgical planning on CT scans) but also for the actual patient-specific functionality of each of these branches.

In the present work, a numerical investigation was performed in a cohort of patients who underwent pulmonary lobectomy in different lobes to characterize the alterations in airways fluid dynamics caused by the surgery. We hypothesize that the proposed approach can effectively: a) describe fluid dynamics parameters (velocity, pressure, wall-shear stress) in the presence of an accurate geometry reconstruction, both in the whole airway tree and at the site of surgery, pre and post-surgery; b) quantify the variation of lobar ventilation between the pre and post-operative models according to the different sites of resection; c) use the pre-operative information provided by the CFD approach to predict the post-operative forced expiratory volume in 1 s (postFEV1CFD) and compare it with the current clinical algorithm (postFEV1anatomic) and the actual post-operative PFTs measured on the patient after the surgery (postFEV1).

## Results

### Study design

Among the 12 selected patients (preFEV1 = 97 ± 16%, postFEV1 = 64 ± 18%)., 7 (58%) were female, and 5 (42%) were male. The mean age was 69.8 ± 10.5 years. Table 1 reports additional information on the study population.

**Table 1:** Study population. For each patient, the location of the lobectomy site, sex, age and clinically measured forced expiratory volume in the 1st second (FEV1) before (preFEV1) and after (postFEV1) the surgery is reported.

ID	Site	Sex	Age	preFEV1	postFEV1
1	RUL	M	78	71.00	43.00
2	RUL	M	79	131.00	107.00
3	RUL	F	70	101.00	88.00
4	RLL	F	76	89.00	67.00
5	RLL	F	79	90.00	55.00
6	RLL	M	72	109.00	64.00
7	LUL	F	49	100.00	58.00
8	LUL	F	52	86.00	44.00
9	LUL	M	74	97.00	70.00
10	LLL	M	56	108.00	71.00
11	LLL	F	78	105.00	49.00
12	LLL	F	74	74.00	51.00

### Mesh selection

**Figure 1:**
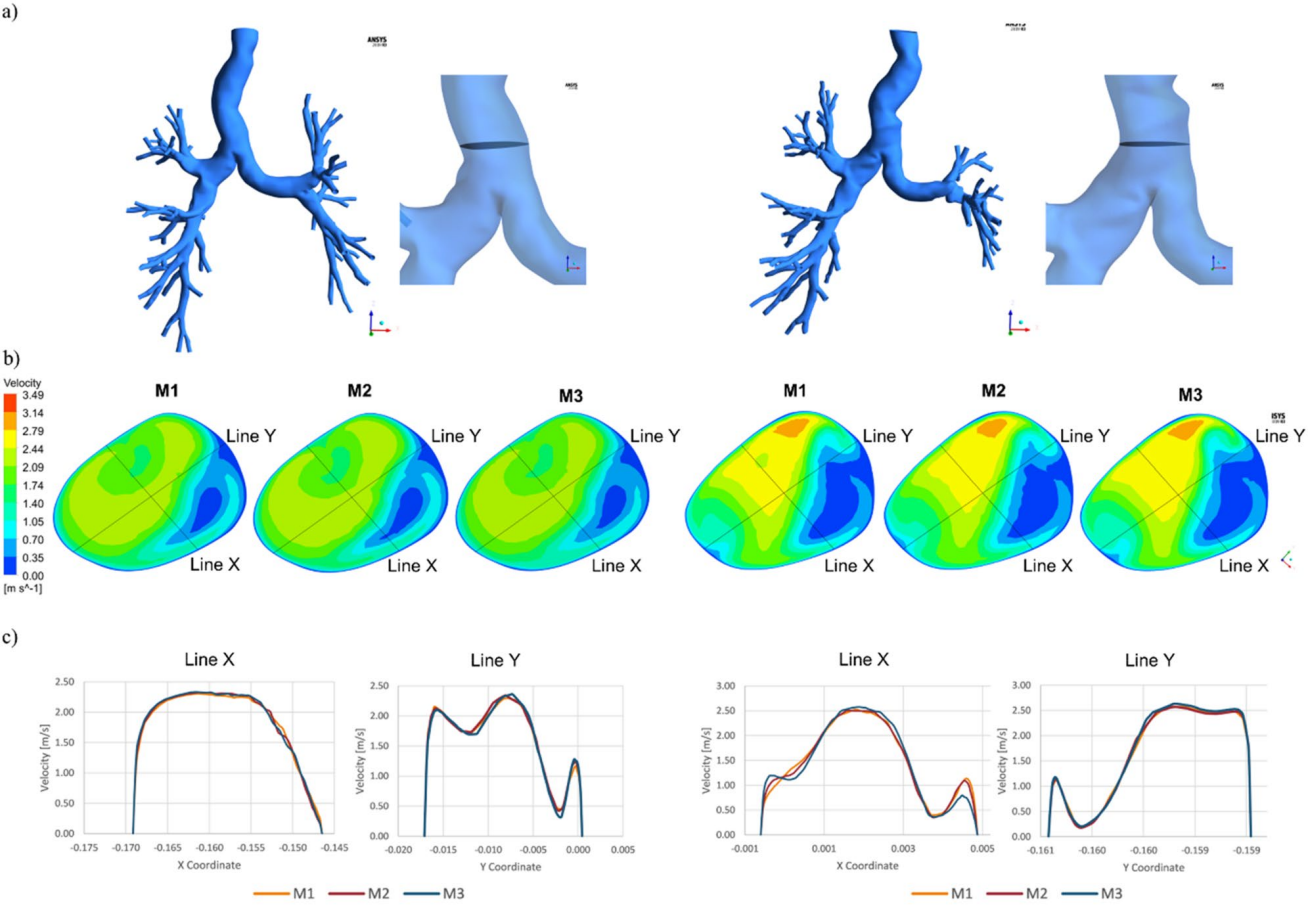
Mesh convergence analysis for a representative left upper pulmonary lobectomy subject (subject 9) in the pre-operative (left) and the post-operative (right) models. In the pre-operative models, for this representative patient, the number of elements for M1, M2, M3 of was equal to 3.7 × 10^6^, 6.3 × 10^6^ , 10.0 × 10^7^, respectively. For the post-operative models M1, M2, M3 were characterized by 3.2 × 10^6^ , 5.8 × 10^6^ , 9.9 × 10^6^ elements, respectively. (**a**) position of the plate inside the bronchial tree. (**b**) contours of velocity on the plate for M1, M2, M3 in the pre (left) and post-operative (right) models. (**c**) Velocity profiles along lines X and Y for the pre (left) and post-operative (right) cases (Fluent v16, CFD-Post v16-Ansys, www.ansys.com).

The convergence analysis showed no relevant differences between the three meshes as reported in Fig. 1c for a representative subject in the pre and post-operative models. Consequently, the meshes with the lowest number of elements were adopted. The average number of elements of the mesh was 3.2 × 10^6^ (± 0.5 × 10^6^) and 3.6 × 10^6^ (± 1.1 × 10^6^) for the pre and post-operative models, respectively. The average skewness was equal to 0.87 ± 0.02.

### Morphological alterations

**Figure 2:**
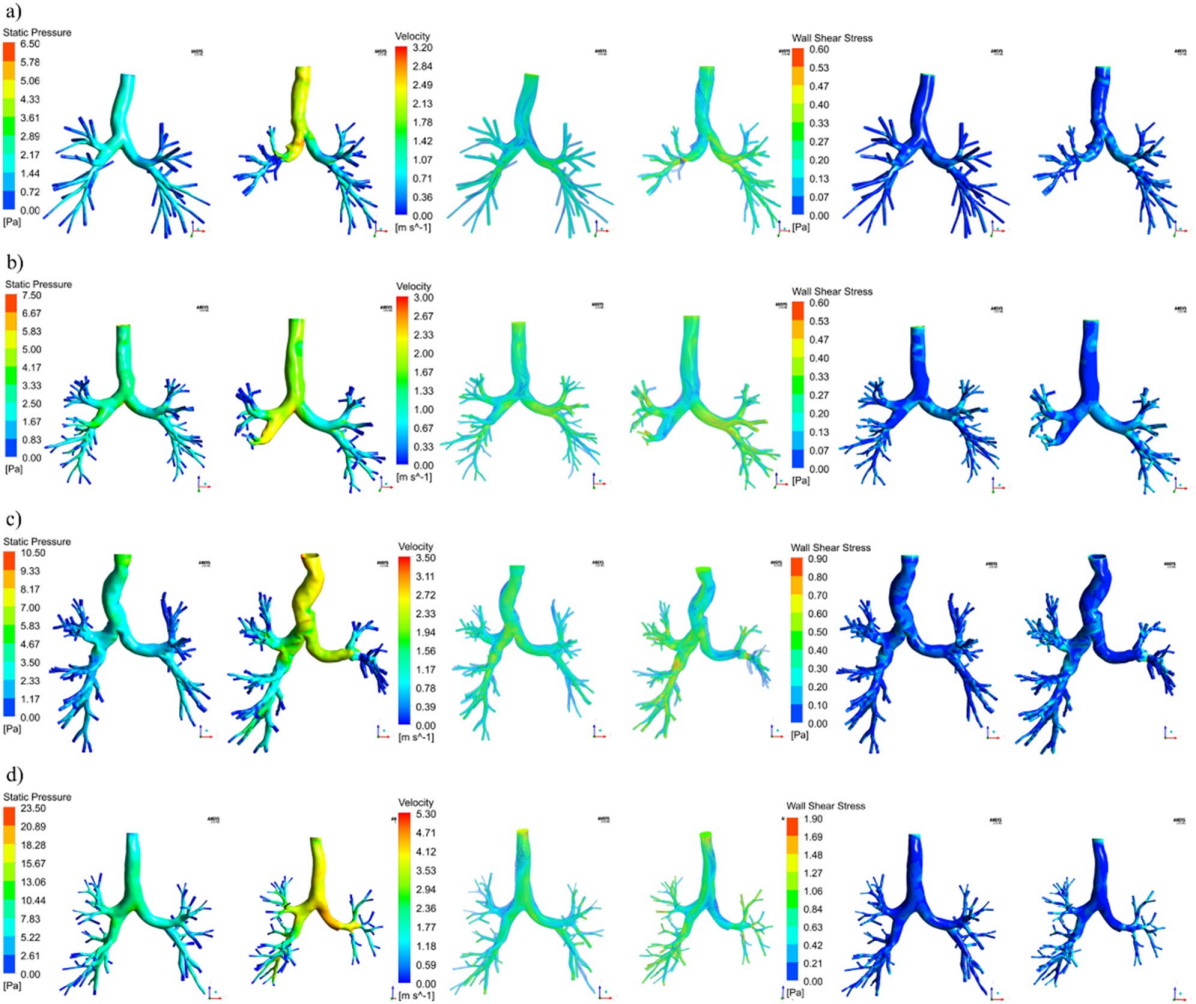
Contours of wall pressure (left), velocity streamlines (middle), contours of wall shear stress (right) on the global models for four representative subjects. (**a**) subject 2, right upper pulmonary lobectomy; (**b**) subject 6, right lower pulmonary lobectomy; (**c**) subject 9, left upper pulmonary lobectomy; (**d**) subject 12, left lower pulmonary lobectomy (Fluent v16, CFD-Post v16-Ansys, www.ansys.com).

The values of the right (θ_R_) and left (θ_L_) in-plane bifurcations angle between the trachea and the main bronchi and the cross-sectional area (CSA) of the remaining lobar segment adjacent to the resection site measured in the middle point of the segment and perpendicular to the centerline are reported in Table 2 for the pre and post-operative models, divided according to the different sites of lobectomy. We observed specific trends in upper lobectomies (both RUL and LUL): the angle between the trachea and the main bronchi on the operated side tends to decrease while showing a mild increase on the contralateral side. Increments of θ_R_ were also observed in RLL lobectomies with an average increase of 10.94 (7.72–13.54) %, while θ_L_ showed mild variations. None of the differences in θ_R_ and θ_L_ between the pre and post-operative models were statistically significant. For both the RUL and LUL lobectomy, the secondary bronchi are twisted upward and severely bent downstream of the suture zone, as can observed in Fig. 2a and Fig. 2c. The CSA of the adjacent segment in the post-operative models resulted significantly lower than the pre-operative ones (p = 0.01) for all the subtypes of lobectomies considered. In general, higher reductions were observed for upper lobectomies: the median decrement was equal to 21 (18–28) % and 34 (26–43) % for RUL and LUL lobectomies, respectively. Excluding patient 11, lower lobe lobectomies showed a cross-sectional reduction of 0.50 (0.40–3.60) %. For subject 11, marked cross-sectional variations were observed with a value equal to -86%. Results concerning this subject are detailed in the Supplementary Material Section. In LLL and RLL lobectomy cases, the geometrical alterations of the remaining lobes appeared to be less marked. No evidence of bronchial stenosis was observed in the remaining lobar segments. Moderate deformations of the RML and of the lingula were observed for RLL and LLL lobectomies cases, respectively.

**Table 2:** Measured values of θ_*R*_, θ_*L*_, and CSA in the pre and post-operative cases. The percentual variation with respect to the pre-operative case is also reported.

ID	Site	θ_R_ [°]		∆θ_R_ [%]	θ_L_ [°]		∆θ_L_ [%]	CSA [mm^2^]		∆CSA [%]
		PRE	POST		PRE	POST		PRE	POST	
1	RUL	143.24	141.62	−1.13	137.13	145.23	5.91	86.89	71.07	−18.21
2	RUL	141.38	139.55	−1.29	127.92	134.61	5.23	149.46	117.56	−21.34
3	RUL	137.59	129.21	−6.09	132.09	141.61	7.21	77.20	55.36	−28.29
4	RLL	141.86	157.38	10.94	128.03	129.57	1.20	34.53	32.28	−6.52
5	RLL	144.72	164.31	13.54	109.27	108.31	−0.88	32.86	32.73	−0.40
6	RLL	126.86	136.65	7.72	125.78	122.61	−2.52	66.88	66.48	−0.60
7	LUL	164.20	161.04	−1.92	134.85	134.86	0.01	42.30	31.25	−26.12
8	LUL	140.07	135.15	−3.51	135.33	136.99	1.23	82.43	47.14	−42.81
9	LUL	141.82	139.52	6.97	123.06	122.03	−0.84	97.78	64.15	−34.39
10	LLL	146.78	155.91	6.22	134.55	133.74	−0.60	104.89	104.45	−0.42
11	LLL	132.51	133.76	.94	126.26	132.43	4.89	86.68	11.72	−86.48
12	LLL	137.59	135.36	−1.62	129.57	133.87	3.32	65.45	58.57	−10.51

### Fluid dynamics parameters

**Figure 3:**
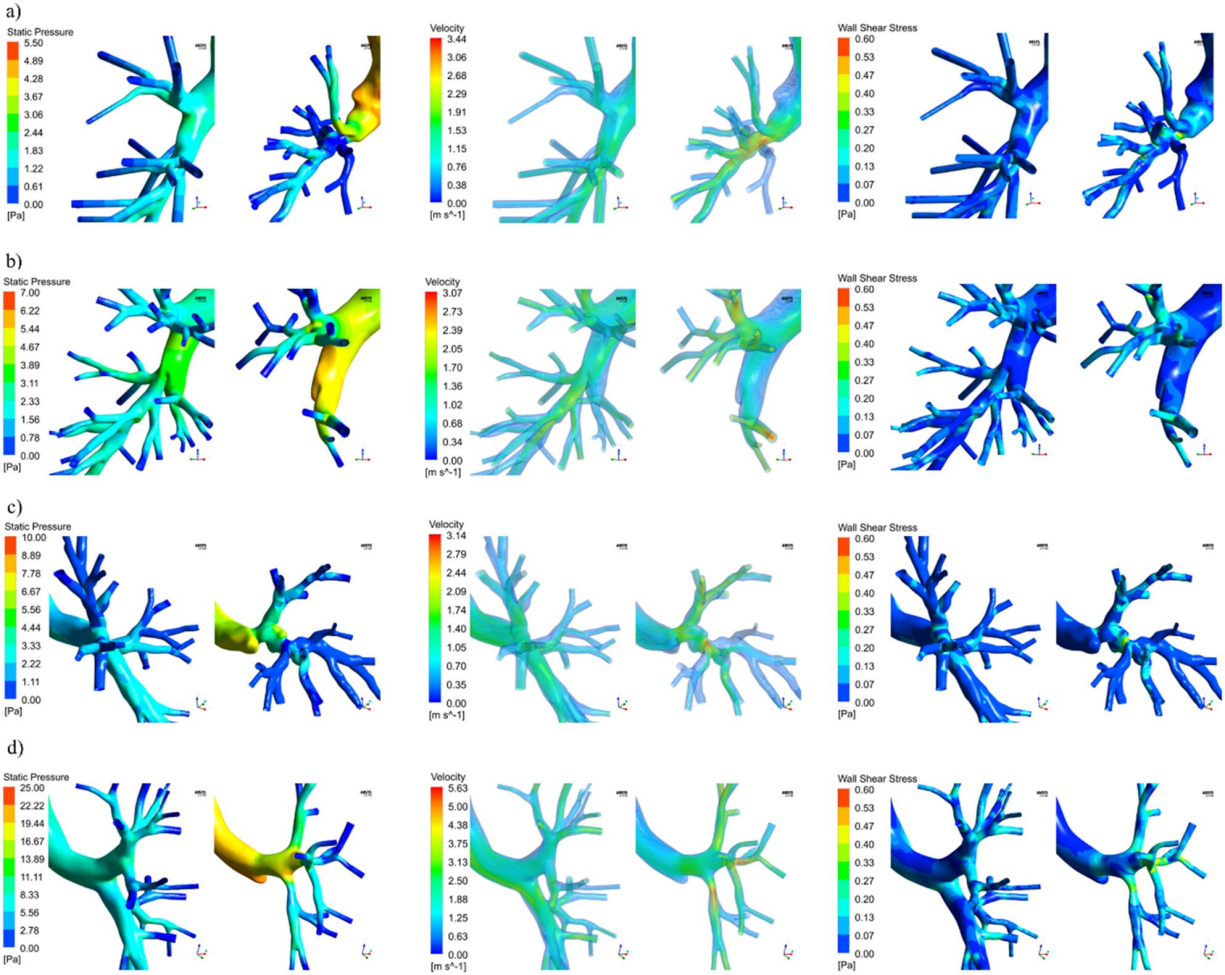
Contours of wall pressure (left), velocity streamlines (middle), contours of wall shear stress for four representative subjects in the proximity of the surgery site. (**a**) subject 2, right upper pulmonary lobectomy; (**b**) subject 6, right lower pulmonary lobectomy; (**c**) subject 9, left upper pulmonary lobectomy; (**d**) subject 12, left lower pulmonary lobectomy (Fluent v16, CFD-Post v16-Ansys, www.ansys.com).

Globally and regardless of the resected lobe, the post-operative configurations showed, given the same inlet flow, an increment in the magnitude of velocity, wall pressure, and wall shear stress for all 12 patients. The peak values of the variables of interest are reported in Table 3. In the comparison between the fluid dynamics parameters in the pre and post-operative models, statistically, significant differences were observed between the peak values of pressure (p < 0.001), velocity (p = 0.0017), and wall shear stress (p < 0.001). In terms of pressure, the maximum value for the pre and post-operative conditions was equal to 14.15 (6.79 – 19.5) Pa and 23.11 (10.56–37.97) Pa with a median increment equal to 8.71 (2.91–17.98) Pa and a correspondent percentual variation equal to 63 (36–102) %. The entire post-operative geometries were subjected to higher pressure (Fig. 2, left). In general, the pressure is higher at the trachea and the main bronchi level, and it progressively settled down in the vicinity of the outlets in both geometries due to the uniform boundary condition applied. For all the subjects under analysis, the global peak of pressure was identified at the level of the carina. Subjects 4 and 11 resulted in the highest value of pressure variation, as detailed in Supplementary Material Section. Locally, peak pressure values were identified at the level of the resection site and at the level of the trachea when bending occurred in the post-operative geometries. The maximum velocity value was equal to 4.31 (2.58–4.84) m/s in the pre-operative configurations and 5.12 (3.19–7.57) m/s in the post-operative ones. The percentual increment in the post-operative cases was equal to 29 (15–52) %. The peak value of velocity was identified at the level of the trachea or main bronchi. However, in upper lobe lobectomies, the severe reduction of CSA in the bronchus downstream the resection site determined a high-velocity magnitude comparable to the global peak for two subjects. A generalized worsening of the dynamic air conditions could be observed in post-operative airways of all the subjects under analysis (Fig. 2, middle), with greater velocity values propagating up to the smallest branching generations. The presence of high-velocity gradients determined wider regions of high wall shear stress (Fig. 2, right) with a median percentual increment of 70 (33–104) %. Global peaks of wall shear stresses generally occurred at the first two generations of the tree, although for LUL and RUL lobectomies, comparable values were found in the first bronchial generation downstream of the suture. Finally, also the pressure drop between the inlet and the outlets significantly increased (p < 0.001) in the post-operative models. Significant negative correlations were identified between the postoperative CSA of the remaining segment and the maximum values of pressure (r = -0.881, p < 0.001), velocity (r = 0.860, p = 0.004), wall shear stress (r = 0.697, p = 0.012). The lower the CSA, the higher the pressure drop (r = -0.888, p < 0.001). No correlation was identified between the values of the θ_R_ and θ_L_ and the fluid dynamic parameters. In Fig. 3, the local comparison of the fluid dynamics on the surgery site for four patients representative of each site of lobectomy is considered. Local maxima of pressure, velocity, and wall shear stress were present at the resection site level and in correspondence to the remaining lobar segments for all the patients under analysis. These local maxima were more marked for those patients who underwent an upper lobe lobectomy and presented evidence of cross-sectional reduction at the levels of the remaining secondary bronchi and severe rearrangements of the remaining lung structures as previously described. For RLL and LLL lobectomy cases, local increment of pressure, wall shear, and velocity were still observable despite the absence of marked geometrical alteration due to the redistribution of flow in the upper lobes. Interestingly, consequences of the lobectomy were also observable on the contralateral side of the tree, where no surgical intervention was performed. Higher velocity, pressure, and wall shear stress values were found independently from the lobectomy site. In one patient (#5, reported in the supplementary material), whose trachea assumed a deformed configuration after surgery, local maxima were also present. No statistically significant differences in terms of percentual variation of pressure, velocity, or wall shear stress were observed for the patients under analysis if grouped for the affected lung (right/left) or position (upper/lower).

**Table 3:** Maximum values of wall pressure, wall shear stress, velocity and pressure drop in the pre and post-operative cases. The respective variations with respect to the pre-operative values are also reported.

ID	Site	P [Pa]		∆P [%]	V [m/s]		∆V [%]	WSS [Pa]		∆WSS [%]	P DROP [Pa]		∆P DROP [%]
		PRE	POST		PRE	POST		PRE	POST		PRE	POST	
1	RUL	15.55	20.21	+ 30	4.79	4.98	+ 4	1.64	1.78	+ 10	15.13	17.12	+ 13
2	RUL	3.75	6.41	+ 71	2.06	3.11	+ 51	0.39	0.60	+ 54	1.67	5.07	+ 203
3	RUL	20.20	36.23	+ 79	4.72	7.63	+ 62	1.24	2.33	+ 89	16.72	32.83	+ 96
4	RLL	19.80	41.12	+ 108	4.67	7.78	+ 67	1.32	2.51	+ 90	16.33	49.43	+ 203
5	RLL	12.68	26.00	+ 105	5.26	7.00	+ 33	1.58	2.93	+ 86	14.05	28.01	+ 99
6	RLL	7.19	11.25	+ 56	2.55	2.98	+ 17	0.39	0.60	+ 54	4.90	5.99	+ 22
7	LUL	19.91	38.55	+ 93	4.86	7.39	+ 52	1.28	2.68	+ 110	13.88	42.50	+ 206
8	LUL	9.18	11.82	+ 29	3.66	3.99	+ 9	0.83	0.98	+ 19	7.58	9.85	+ 30
9	LUL	6.65	10.33	+ 55	2.67	3.43	+ 29	0.70	0.88	+ 27	5.38	8.76	+ 63
10	LLL	5.34	6.63	+ 24	2.46	2.81	+ 14	0.51	0.96	+ 89	4.86	5.77	+ 19
11	LLL	18.25	46.00	+ 157	6.64	8.44	+ 27	1.16	2.60	+ 124	12.55	38.81	+ 209
12	LLL	23.10	35.87	+ 55	4.15	5.26	+ 26	1.22	1.88	+ 5	12.00	22.67	+ 89

### VFR lobar distribution

**Figure 4:**
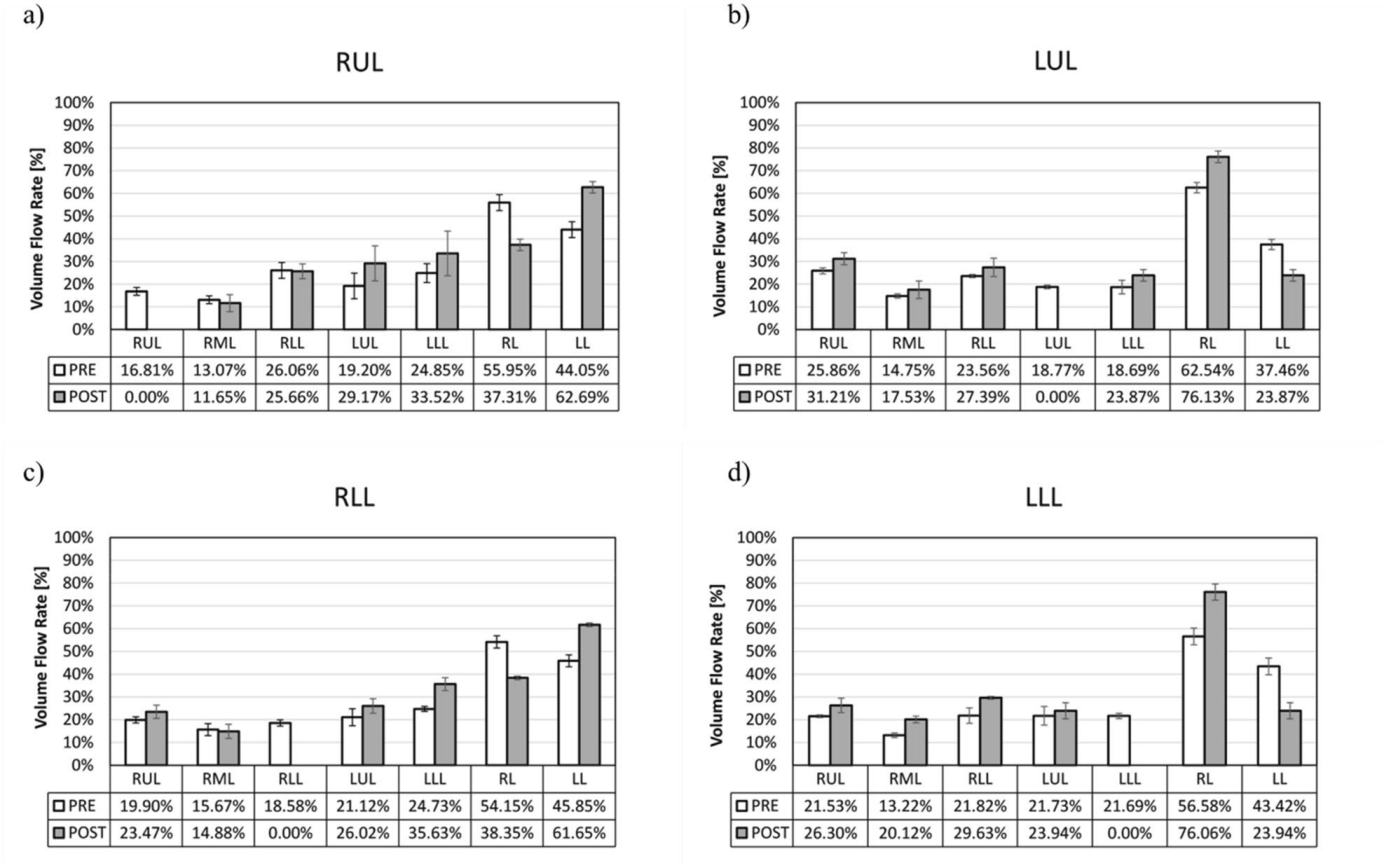
Regional Volume Flow Rate (VFR) in percentage values, estimated through the numerical simulations, for the pre-operative case (white) and the post-operative case (gray) divided according to the lobectomy site . (**a**) right upper lobe (RUL) lobectomies; (**b**) left upper lobe (LUL) lobectomy; (**c**) right lower lobe (RLL) lobectomy, (**d**) left lower lobe (LLL) lobectomy. RML = Right middle lobe.

Figure 4 shows the distribution of VFR for the subjects under analysis grouped according to the site of lobectomy. In the pre-operative models (white bars), the right lung received a higher amount of flow. Lower lobes showed higher flow than the upper ones, and the RML was the lobe constantly receiving less flow. The post-operative models revealed considerable rearrangements of the VFR distribution for all the typologies of lobectomy considered. In the comparison of the overall population, significant differences arose between the pre and post-operative models for both the operated lung (p < 0.001) and the contralateral lung (p < 0.001). However, if the lobe to be removed is neglected in calculating the VFR to the operated lungs, the difference between the pre and post-operative configurations is not significant (p = 0.121). Finally, in the post-operative models, a statistically significant imbalance was found between the operated and the contralateral lung with a significant increment (p < 0.001), in the contralateral lung. This was not observed in the pre-operative comparison between the lung affected by the tumor and the contralateral one (p = 0.384). Table 4 summarizes the value of the clinically measured FEV1 and the estimated post-operative function with both the anatomical formula and the CFD approach. The FEV1 estimation through CFD (Eq. (2.4)) showed a significant correlation with the reference clinical estimation through Eq. (2.3) (r = 0.95, p < 0.001), and with the actual clinically measured post-operative FEV1 (r = 0.806, p = 0.002). For the subjects under analysis, both the CFD method and the anatomical formula tend to over-predict the clinically measured FEV1 after the surgery.

**Table 4:** Values of the clinically measured FEV1 after the lobectomy surgery (postFEV1) and the estimated post-operative function with the anatomical formula (postFEV1_anatomic_) and the CFD approach (postFEV1_CFD_).

ID	Site	postFEV1	postFEV1_anatomic_	postFEV1_CFD_
1	RUL	43	60	59
2	RUL	107	110	112
3	RUL	88	85	82
4	RLL	67	66	71
5	RLL	55	66	73
6	RLL	64	80	91
7	LUL	58	74	81
8	LUL	44	63	69
9	LUL	70	71	80
10	LLL	71	85	84
11	LLL	49	83	81
12	LLL	51	58	58

## Discussion

In the present work, CFD was applied to evaluate the post-operative fluid dynamic alterations following pulmonary lobectomies performed in different lobes. Patients' specific geometries were reconstructed from CT data, the airflow simulated, and the results analyzed to identify fluid dynamics parameters and VFR variations. Computational results were used to estimate the post-operative function (postFEV1CFD), which was compared against the current clinical standard algorithm (postFEV1anatomic) and the actual post-operative measurements (postFEV1). The present study allowed for a more quantitative assessment of the pre-operative condition and an enhanced understanding of the post-operative alterations. The information of pre-operative flow rate can be used, according to Eq. (2.4), to estimate the post-operative function and provide additional information concerning patient operability without the actual need for post-operative data. In contrast to the post-operative estimation performed with the anatomical formula, CFD accounts for local fluid dynamics characteristic of each branch that will eventually be resected during the surgery.

Regarding the numerical simulations' settings, it was chosen to simulate a steady-state peak inspiratory condition. The choice of a steady-state simulation was a reasonable simplification considering the results related to calculating the Womersley number and the Strouhal number for the models under consideration. The evaluation of the inspiration condition, related to the study's retrospective nature, enables the evaluation of higher mechanical stimuli occurring in the airway walls with respect to the expiratory condition^25,26^. A uniform pressure was set at the outlet boundaries not to constrain the airflow partition and the lobar flow rates^15^. The k-ω SST model has been widely adopted for airflow simulation in the airways, and it has been demonstrated to provide an optimal trade-off between the accuracy of the prediction of low turbulence flow in the respiratory system and the availability of computational resources^15,23,27^. The choice of considering the air as a Newtonian fluid at ambient conditions is motivated by the fact that the Mach number (the ratio between the velocity of air and the speed of sound) in the airways is approximately 0.3 and that temperature and humidity variations within the physiological range of the respiratory system cause negligible variations on the air properties with respect to environmental conditions^28^. Finally, the hypothesis of rigid walls, well documented in the literature^29–32^, can be considered acceptable due to the relatively high percentage of cartilage in the airway tissue of the central airways^33^.

In terms of geometrical characterization of the pre and post-operative models, we observed severe alterations of the remaining pulmonary structures in the case of upper lobectomies. Although present, the structural modifications were less relevant for lower lobe lobectomies and mostly limited to the RML and the lingula shift to the vacant area, in accordance with previous studies^34^. The alterations were also relevant at the trachea and main bronchi level in two subjects, as described by previous studies^24,35^. The significant decrement of the cross-section downstream the surgery site is confirmed by studies for the LUL cases^23,24^, and the results of the proposed work suggest that this consideration is also verified in the RUL lobectomies cases. Lower lobe lobectomies, on average, showed lower cross-section variations with the only exception of subject 11. As reported in the supplementary material section, the matching with the clinical course revealed that the patient had suffered from pneumonia with pleural effusion. Our analysis readily detected this abnormal clinical course, and the results were widely different from other cases. Indeed, if this subject is neglected, the median reduction of CSA in the case of lower lobectomies is equal to 0.60 (0.40–5.53). In contrast to Gu et al.^23^, no systematic increment of θ_R_ and decrement of θ_L_ was observed in the LUL post-operative cases, possibly because of the limited size of our population. Although the characterization and the role of structural modifications on post-operative pulmonary function is still an open field of research^36^, the pre and post-operative values measured angles in our study are in line with the expected intersubject variability, also accounting for the sex-related differences between women and men^37^.

The numerical simulation results showed that, under the same inlet flow rate, increased mechanical stimuli emerged in the post-operative models with increased velocity, pressure, and wall shear stress in the whole bronchial tree. These fluid dynamic alterations are derived from the resection of an entire lung lobe and the geometrical adaptation of the remaining structures, especially for upper lobe lobectomies. Limited to LUL lobectomies, our results are consistent with the two previous studies characterizing this type of lobectomy^23,24^. With respect to Gu et al.^23^, relatively higher variations of fluid dynamic parameters are observed in our cohort of patients that underwent LUL lobectomy, although this can be possibly ascribed to the higher inlet flow rate considered in the simulations of the presented work. The obtained values are indeed in line with Tullio et al.^24^, who considered the same inlet flow of the presented study. For upper lobes lobectomies, the increment of pressure, velocity, and wall shear stress can be associated with the severe bronchial stenosis observed and the general re-adaptation of the remaining lower lobes. For lower lobes lobectomies, despite the presence of mild stenosis of the bronchi upward the suture site, it seems more likely that the fluid dynamics variations are associated with the redistribution of the flow that physiologically is much higher the lower lobes than to the upper ones. The increment in pressure drop observed after the surgery, independently from the lobectomy site, can suggest a higher fluid dynamic resistance to airflow and a consequent pressure-driven increase during inspiration^18,38,39^. The result is consistent with previous computational studies on lobectomy^23,24^. The quantitative and local information on fluid dynamics parameters provides a better understanding of the alteration induced by the re-adaptation of remaining lung structures and has a physiological and clinical impact. Indeed, the increment in the values of wall shear stress caused by the increased local velocity at the level of the surgical site can impact the endothelial cells of the bronchial tissue^40^, and represent a risk especially in case of higher inlet flow rates, for example during exercise conditions.

In terms of flow distribution, the results in the pre-operative models are consistent with previous airflow dynamics and particle deposition studies. The right lung receives more flow due to its bigger size if compared to the left one^41,42^, although, as expected, this difference is not statistically relevant. The lower lobes receive a greater portion of inhaled flow rate with respect to the upper lobes, while the RML, being the smallest lung lobe, receives the lowest amount of flow rate^15,17,43^. In the post-operative models, a significant increment in the contralateral lung flow with respect to the operated lobe was observed, as confirmed by previous computational studies limited to the LUL lobectomies cases^23,24^. The presented findings suggest that this trend is also confirmed for RUL, LLL, and RLL resections. However, contrary to Gu et al.^23^, the remaining structure in the operated lung does not seem to undergo statistically significant variations in the inflow, as in LUL lobectomies. This supports the idea that the contralateral lung tends to undergo the major flow variations and takes most of the flow physiologically meant for the resected lobe. These findings are supported by clinical studies on pulmonary function after lobectomy, which evidenced a reduction in volume variations and regional ventilation in the remaining lobes of the operated lung^44,45^.

Morphological re-adaptations and the mediastinum shifting, make the measurement of lobar ventilation distribution through functional imaging particularly challenging in pulmonary lobectomy surgery^44^. CFD can therefore provide clinically relevant support to address this issue. In addition, the possibility to obtain patient-specific information on fluid dynamics and lobar ventilation could become a valuable asset in the prescription of personalized respiratory physiotherapy for better post-operative management.

The comparison presented in Table 4 among the estimations of the post operative function with the CFD (postFEV1CFD), the anatomical method (postFEV1anatomic) and the clinical measured values (postFEV) showed an overall good consistency. However, it appears that the CFD approach does not currently provide a better estimation of the actual clinical measure. This can be possibly related to the relatively limited number of subjects under analysis and to relatively simplified settings adopted in the numerical simulations, in particular in terms of inlet and outlet boundary conditions.

The proposed work is affected by some limitations deriving from the available input data and the computational resources at disposal. Several studies have demonstrated the efficacy of adopting patient-specific lobar flow rate of the patient as outlet boundary condition^32,46–48^: this requires functional imaging or at least the evaluation of volume variation between inspiratory or expiratory scans. Due to the retrospective nature of the collected data, no expiratory scans were available, as the current clinical follow-ups for these patients include only inspiratory CT images. For this reason, as most authors in literature^15,17,39,49–51^, a uniform pressure at the outlets was applied in the simulations. As an alternative, impedance-based boundary conditions that account for the impedance of the parts of the airway tree that cannot be resolved (either for computational reasons or for intrinsic limitations of the CT), can be applied to provide physiological boundary conditions for flow in the tracheobronchial region^52^. Besides, to better characterize the fluid dynamics in the proximity of the trachea inlet and prevent artifacts related to the application of the boundary condition, it would be beneficial to include flow extension to allow for flow development or directly imposing developed profile at the inlet.

No direct validation of the results related to CFD was performed on these patients. Nevertheless, the proposed numerical approaches have been validated and have been demonstrated to reasonably agree with functional measurements from imaging (e.g., in Luo et al.^15^) and to more complex models such as DNS^26^. In addition, although consistent with previous literature, the assumption of a steady-state solver neglects possible unsteadiness. Finally, in our analyses, only the limit case peak inspiratory flow under normal breathing was considered because it was expected to provide the highest mechanical stimuli in the airway walls^25,26^. Future directions of development will include the adoption of a dynamic inlet flow rate mimicking a physiological-like breathing waveform^48^ and the investigation of different breathing conditions during exercise or mechanical ventilation, including the actual breathing waveform of the patients or flow rate data from literature^45^. Unsteady simulations can also be performed at the same inlet flow rate to account for unsteadiness. Prospective studies on a selected cohort of patients, including expiratory scans and functional imaging can be designed both to apply patient-specific boundary conditions and to validate the numerical solution on the specific dataset. In addition, it would be possible to perform the CFD analysis of the expiratory phase. Ideally, the acquisition protocol should also include the patient's upper airways for a more realistic representation. Also, to better understand the post-surgery remodeling, the geometrical characterization of the airways in the pre and post-operative conditions can be further developed with more comprehensive metrics such as the regional volumetric changes and the evaluation of different cross-sections along the bronchial tree. Finally, an extended cohort of subjects, including patients who underwent middle lobe lobectomies and bilobetomies, should be analyzed. Finally, an extended cohort of subjects, including patients who underwent middle lobe lobectomies and bilobetomies, should be analyzed. The inclusion of more patients, together with the evaluation of more complex numerical modelling approaches, would also be highly beneficial in the assessment of the degree of accuracy of post-operative function estimation through the CFD approach compared to the current clinical algorithm.

## Conclusions

A CFD approach to evaluate the consequences of post-operative anatomical alterations on airflow dynamics in the airways model of patients that underwent pulmonary lobectomy of different lobes has been proposed. The airflow disturbances were quantified both globally and locally. To the best of our knowledge, this is the first study that applies CFD to subjects who underwent lobectomies in different sites. The results highlighted that alterations related not only to the resection of an entire lobar structure but also to the subsequent re-adaptation of the remaining lung structures. The regional flow rate to the lobes was computed, suggesting that compensatory effects occur at the level of the contralateral lung rather than in the remaining operated lung lobe(s). The estimation of the post operative function from the simulations (postFEV1CFD) was comparable with the results of the current clinical algorithm (anatomical calculation, postFEV1anatomic) and the actual clinical data (postFEV1). This approach will provide additional information concerning patient operability, accounting for actual patient-specific morphology and functionality alterations and for local fluid dynamics characteristic of each branch that will eventually be resected during the surgery.

## Methods

### Study design

This retrospective study was approved by the Fondazione IRCCS Ca' Granda – Ospedale Maggiore Policlinico of Milan institutional review board (approval number: 2583), and informed consent was obtained from all patients. All methods were carried out in accordance with relevant guidelines and regulations***.*** A query of the institution's radiology database for chest CT images acquired for clinical purpose in breath-hold at full inspiration one month before and six months after surgery was performed for early-stage lung cancer patients, undergoing pulmonary lobectomy, aged > 18 years old. A subgroup of CT examinations, which presented technical acquisition parameters suitable for the analysis, were selected for the study. All identifying information was removed from the CT images before the analysis. Clinical details related to the transplantation and spirometry results were obtained through the electronic medical record. Exclusion criteria were middle lobectomy, bilobectomy, CT performed more than one month before or more than six months after surgery, patients with no clinical details available both before and after surgery. Chest CT images acquired at Thoracic Surgery and Lung Transplantation Unit of Fondazione IRCCS Ca' Granda – Ospedale Maggiore Policlinico of Milan, Italy.All methods were carried out in accordance with relevant guidelines and regulations. The following CT scan (SOMATOM Definition dual-source CT; Siemens, Forchheim, Germany) settings were adopted during image acquisition: tube voltage = 100–120 kV; tube current = 142–617 mA according to body mass index, matrix size = 512 × 512, slice thickness = 0.62–1 mm, in-plane resolution = 0.62–0.90 mm; axial resolution = 0.60–1.0 mm. Reconstruction was obtained with soft kernels (B30f., B31f.).

Spirometry, including forced vital capacity (FVC) and forced expiratory volume in 1 s (FEV1), were collected before and after the surgery, at the time of CT, and reported in terms of percentage predicted of normal values. The surgery was carried out in the period from January 2015 to June 2019. We divided the study population into 4 groups, one for each type of lobectomy: right upper lobectomy (RUL), right lower lobectomy (RLL), left upper lobectomy (LUL), left lower lobectomy (LLL). We consecutively selected 12 subjects meeting the inclusion/exclusion criteria, up to reach 3 complete cases in each of the groups.

### Generation of the airway model

The 3D subject-specific airway tree models for the pre-operative and post-operative configurations were semi-automatically segmented in Mimics (Materialise NV, Belgium). The geometrical reconstructions were processed with 3-Matic (Materialise NV, Belgium) and ANSYS SpaceClaim (ANSYS Inc., Pennsylvania, USA) and export to ANSYS Fluent for meshing. The average volume of the reconstructed geometries was equal to 5.97 × 10^4^ (± 2 × 10^4^) mm^3^ and 5.43 × 10^4^ (± 1.99 × 10^4^) mm^3^ for the pre and post operative geometries respectively.

### Mesh generation and convergence analysis

A tetrahedral unstructured grid type was chosen due to the geometrical complexity. To resolve fluid properties in the region proximal to the wall, 15 inflation layers were created. The first layer thickness was set equal to 0.01 mm, corresponding to an estimated y^+^  < 1. The quality of the generated mesh was evaluated through the maximum skewness criterion (equilateral volume skewness < 0.90). Three mesh refinements (M1, M2, M3) were generated for each geometry. The total number of elements for each mesh varied according to the complexity of the patient geometries. Over the whole population, for the pre-operative cases, the average number of elements was equal to 3.2 × 10^6^ (± 0.5 × 10^6^), 5 × 10^6^ (± 1.1 × 10^6^), 9.3 × 10^6^ (± 2.4 × 10^6^) for M1, M2, M3, respectively. For the post-operative cases, the average number of elements was equal to 3.6 × 10^6^ (± 1.1 × 10^6^), 5.7 × 10^6^ (± 2.1 × 10^6^), 10.0 × 10^6^ (± 3.9 × 10^6^) for M1, M2, M3, respectively. The convergence analysis was conducted considering the relative variations between the meshes regarding volume flow rate (VFR) at the outlets, with a tolerance inferior to 0.5%. Moreover, the local velocity profile was evaluated for the three meshes at a cross-sectional plane at 2 cm distance from the carina (Fig. 1a). Two axes, mutually rotated at 90° from each other, were chosen to plot the profiles as shown in Fig. 1b.

### Airflow simulation

The influence of oscillatory behavior on the flow was preliminarily investigated. The effects of unsteadiness on the average flow characteristics, computed through a steady solution, are modest if the Womersley (Wo) and the Strouhal (S) numbers are lower than 10 and 1, respectively^41^. The Womersley number, i.e., the ratio of unsteady forces to viscous forces, is calculated as:

2.1 $$Wo= \frac{D}{2}{\left(\frac{2\pi f}{\upsilon }\right)}^{0.5}$$

The Strouhal number, i.e., the ratio of unsteady forces to inertial forces, is calculated as:

2.2 $$S= \frac{2\pi fD}{\overline{u} }$$

where *f* is the breathing frequency, *υ* is the air kinematic viscosity,* D* is the hydraulic diameter of the trachea, and $\overline{u }$ is the average velocity at the corresponding diameter. In the cases under analysis, *f* = 15 breaths per minute and *υ* = 1.79 × 10^–5^ kg/ms were assumed. *D* was measured at the inlet cross-section for all the pre and post-operative models, while the velocity at the trachea was obtained by dividing the inlet flow rate by the inlet cross-section. The inspiratory flow was assumed to equal 0.5 L/s^53^, simulating the peak inspiratory flow during quiet breathing. Under those conditions, the average Womersley number was equal to 2.45 (± 0.35), and the average Strouhal number was equal to 0.012 (± 0.005). The Womersley and Strouhal numbers were equal to 2.38 (± 0.37) and 0.011 (± 0.005) for the post-operative cases. Consequently, steady-state simulations were performed. The finite volumes method was adopted for the numerical solution. Since the average Reynolds number was equal to 2606 ± 394 and 2707 ± 457 for the pre and post-operative cases, respectively, turbulence was modeled using the Shear Stress Transport (SST) k-ω model with Low Reynolds Number (LRN) correction with a turbulent intensity of 5% and a viscosity ratio (µ_T_ /µ) of 10. A steady inspiratory flow corresponding to 0.5 L/s was set as a boundary condition at the trachea inlet. A uniform reference pressure was set at each outlet boundary, and a no-slip condition was imposed at the solid walls, considered rigid, stationary, and smooth. The air was modeled as an incompressible Newtonian fluid, with density and dynamic viscosity equal to 1.225 kg/m^3^ and 1.79 × 10^−5^ kg/ms. This is an acceptable assumption for the airflow in the human airways, where the Mach number (the ratio between the velocity of air and the speed of sound) is close to 0.3, and the variation air properties are negligible^28,41^. The hypothesis of rigid walls with the no-slip condition is well documented in the literature^29–32,41^ and can be considered reasonably valid due to the relatively high percentage of cartilage in the airway tissue of central airways^33^. The following settings were adopted: pressure-based solver; a second-order upwind scheme for the momentum equations; Green-Gauss cell-based method for gradients evaluation; and the semi-implicit method for pressure linked equations (SIMPLE) algorithm for pressure–velocity coupling. Convergence was set as residuals less than 10^−6^. Calculations stopped when the residuals converged, and the solution was evaluated to be stable. The results were reported in terms of velocity magnitude, wall pressure, and wall shear stress. The pressure drop was calculated as the difference between the mean pressure at the inlet of the trachea and the mean pressure of the outlets. Finally, the regional ventilation of each lobe was computed for both the pre-operative and post-operative cases by summing the VFR of the corresponding outlets.

### Geometrical characterization

The right (θ_R_) and left (θ_L_) in-plane bifurcations angle between the trachea and the main bronchi were manually measured using the bifurcating nodes of the centreline reference points. The operation was performed on both the pre and post-operative geometries. Additionally, the cross-sectional area (CSA) of the remaining lobar segment adjacent to the resection site was measured in the middle point of the segment. The cross-section perpendicular to the centerline of the airway in the given point was selected.

### Estimation of the post operative function

The estimation of the post-operative function (postFEV1_anatomic_) has been performed in accordance with the current clinical guidelines^9,54^, using the anatomical, segment counting method^10,11^:

2.3 $${postFEV1}_{anatomic}={preFEV1} \times \left(1- \frac{y}{z}\right)$$

where y is the number of functional or unobstructed lung segments to be removed, and z is the total number of functional segments. The total number of segments for both lungs is assumed to be equal to 10 (3 RUL, 3 in the RML, and 4 in the RLL) for the right lung and 9 to the left lung (5 in the LUL, 4 in the LLL).

The estimation of the post operative funtion through the CFD (postFEV1CFD) approach was computed by multiplying the pre-operative function by the remained functionality of the lung estimated as the percentual flow to the remaining lobes, that will not be resected during the surgery (VFR_remaning lobes_).

2.4 $${postFEV1CFD}={preFEV1}\times {VFR}_{remaining lobes}$$

The post-operative function estimated with the anatomical formula (postFEV1anatomic) and the CFD method (postFEV1CFD) was compared against the actual clinical FEV1 (postFEV1) measured from the patients after the surgery.

### Statistical analysis

Study population and mesh element number data are presented as mean ± standard deviation. Values of velocity, wall pressure, walls shear stress, and pressure drop are reported as median (25th percentile–75th percentile) unless stated otherwise. Relative variations were computed with respect to the pre-operative condition as reported in Eq. (2.5):

2.5 $$\Delta X= \frac{Xpost- Xpre}{Xpre}\times 100$$

where X is the parameter of interest.

The assumption of normality was tested through Shapiro–Wilk Test. If normality was verified, paired t-test was adopted to confront data. For not parametric data, Wilcoxon matched pairs test was adopted. Pearson Correlation was used to evaluate correlations. The level of statistical significance was set at P < 0.05.

## Supplementary Information

Supplementary Information.
