# Supplementary material for: Functional analysis of the airways after pulmonary lobectomy through computational fluid dynamics
Source: Sci Rep. 2022 Feb 28;12:3321. doi: 10.1038/s41598-022-06852-x (PMC8885819; doi:10.1038/s41598-022-06852-x)
Supplement: Supplementary file 1 — Supplementary Information. [file 41598_2022_6852_MOESM1_ESM.docx]

Functional analysis of the airways after pulmonary lobectomy through computational fluid dynamics

**Authors**

Lorenzo Aliboni^1*^,

Marta Tullio^1^,

Francesca Pennati^1^,

Antonella Lomauro^1^,

Rosaria Carrinola^2,3^,

Gianpaolo Carrafiello^4,5^,

Mario Nosotti^2,3^,

Alessandro Palleschi^2,3^,

Andrea Aliverti^1^

**Affiliations**

^1^ Dipartimento di Elettronica, Informazione e Bioingegneria, Politecnico di Milano, Italy

^2^ University of Milan, Department of Pathophysiology and Transplantation, Milan, Italy

^3^ Thoracic Surgery and Lung Transplantation Unit, Fondazione IRCCS Ca’ Granda Ospedale Maggiore Policlinico of Milan, Italy

^4^ Diagnostic and Interventional Radiology Department, Fondazione IRCCS Cà Granda Ospedale Maggiore Policlinico, 20122 Milan, Italy.

^5^ Department of Radiology and Department of Health Sciences, Fondazione IRCCS Cà Granda Ospedale Maggiore Policlinico and University of Milano, 20122 Milan, Italy.

**^*^Corresponding Author**

Email: lorenzo.aliboni@polimi.it

Phone: +39 - 3341896755

Address: TBMLab - Laboratorio di Tecnologie Biomediche,

Dipartimento di Elettronica, Informazione e Bioingegneria (DEIB), Politecnico di Milano

Via Colombo, 40 (2° floor), 20133 Milan, Italy

**A Supplementary material**

**A.1 Analysis of the results for subjects 5**

The results of the simulations on subject 5 are reported in figure S1. As highlighted in the results and discussion session of the paper, a marked deformation of the trachea was observed in the postoperative model. As a consequence, local peaks of pressure, velocity and wall shear stress occurred. In terms of θ_R_, θ_L_ and cross-sectional area (CSA), consistently with the other subjects no relevant variations were observed.

**A.2 Analysis of the results for subject 11**

The results regarding subject 11 are reported and discussed in the following paragraphs. In terms of morphological alterations, no relevant variations of values of θ_R_ and θ_L_ between the pre and postoperative models were identified. Overall, angle measurements were in line with the other subjects and with the expected physiological variability. On the contrary, after the surgery, a marked reduction in cross-sectional area (CSA) on the lobar bronchus downstream the suture site is observed with respect to the preoperative model. The reduction (-86%) was the highest among the twelve subjects of the study and it was even more evident taking into consideration that lower lobes lobectomies exhibited a median reduction of 0.60 (0.40-5.53) %. The results of the computational fluid dynamic (CFD) simulations for subject 11 are reported in Figure 1 in terms of wall pressure, velocity, and wall shear stress. It can be observed that this patient is characterized by generally higher values of pressure, velocity and wall shear stress if compared to the other subjects in the study. This can be reasonably ascribed to the reduction in CSA which caused an increment of the pressure upward the suture site and determined a peak of velocity and wall shear stress at the level of the remaining lobar bronchus. Further analysis on this subject confirmed its abnormal postoperative course characterized by the presence of lung inflammation due to a pneumonitis as confirmed also by the CT scans reported in Figure 3. Indeed, subject 11 was also characterized by the highest decline in FEV1 after the surgery (-53% with respect to the preoperative measurement). As reported in the discussion section of the paper, the inflammation of lung tissue determined severe airways narrowing, this can explain the morphological alterations in terms of CSA and the consequent abnormal fluid dynamic behaviour observed.

**Supplement Material –Figure Legends**

**Figure S1**

Contours of wall pressure (a), velocity streamlines (b), contours of wall shear stress (c) on the global models for subject 5. The preoperative and the postoperative models are reported on the left and on the right, respectively (Fluent v16, CFD-Post v16-Ansys, [www.ansys.com](http://www.ansys.com)).

**Figure S2**

Contours of wall pressure (a), velocity streamlines (b), contours of wall shear stress (c) on the global models for subject 11. The preoperative and the postoperative models are reported on the left and on the right, respectively (Fluent v16, CFD-Post v16-Ansys, [www.ansys.com](http://www.ansys.com)).

**Figure S3**

Axial (left), coronal (middle), and sagittal (right) view of the CT scans acquired for subject 11 before (a) and after (b) the surgery.

**Supplement Material – Figures**

**Figure S1**

**
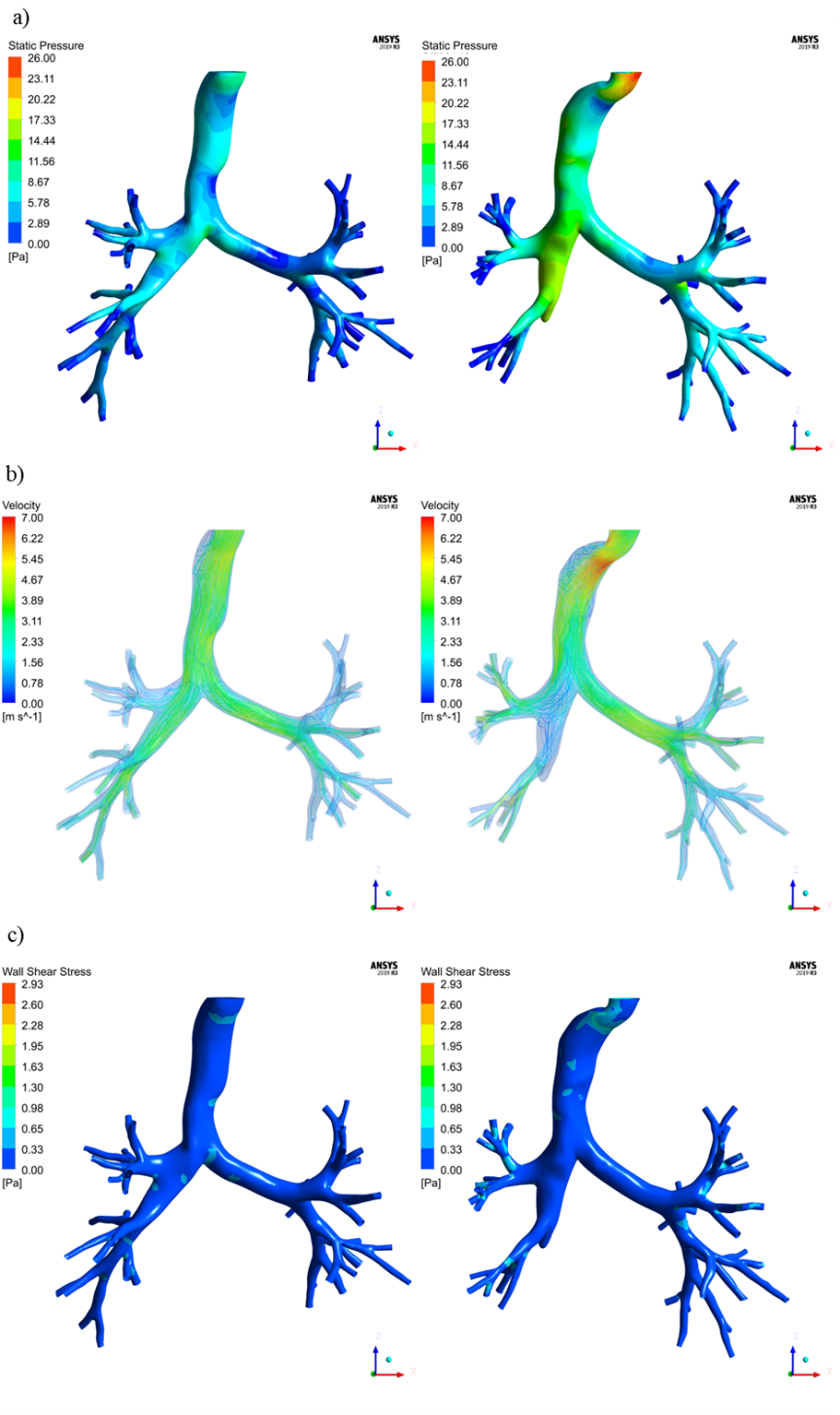
**

**Figure S2**


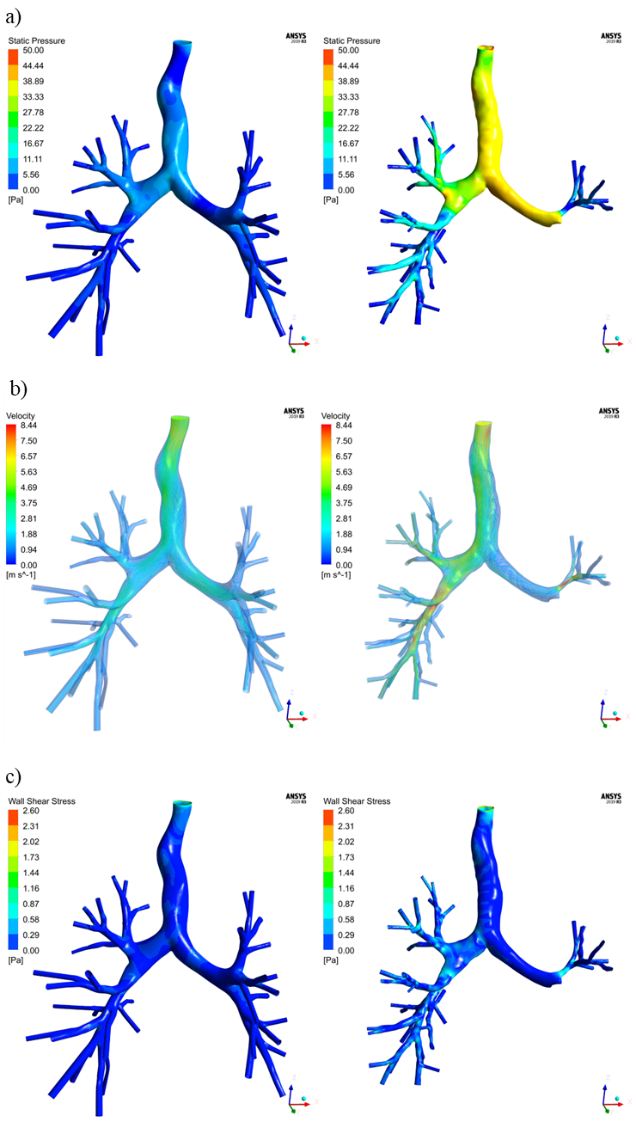


**Figure S3**

**
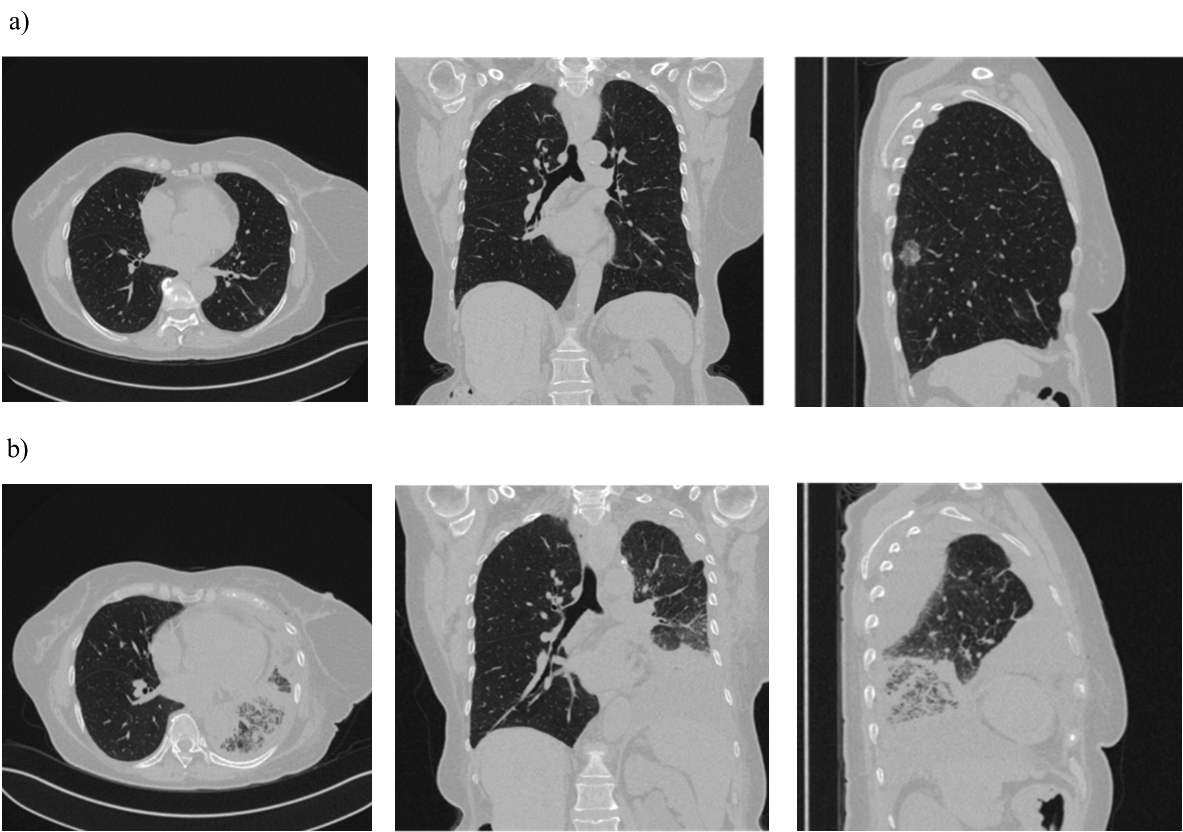
**
